# An Aqueous Fungal-Enzymatic Strategy for the Surface Engineering of Bamboo toward Sustainable High-Performance Biocomposites

**DOI:** 10.1021/acssuschemeng.6c03453

**Published:** 2026-06-30

**Authors:** Yu Qin, Yingjie Wang, Jieyu Yang, Guanben Du, Mizi Fan, Yan Xia, Xiaojian Zhou, Yonghui Zhou, Jingjing Liao

**Affiliations:** † Yunnan Provincial Key Laboratory of Wood and Bamboo Biomass Materials, 12617 Southwest Forestry University, Kunming 650224, China; ‡ College of Engineering, Design and Physical Sciences, Brunel University London, Uxbridge UB8 3PH, U.K.; § College of Materials and Energy, South China Agricultural University, Guangzhou 510642, China; ∥ Yunnan Agricultural University, Kunming 650201, China

**Keywords:** bamboo biocomposites, fungus-secreted enzyme, enzymatic delignification, bioselectivity, interfacial structure and bonding

## Abstract

Bamboo biocomposites hold significant potential for applications in the construction, automotive, and logistics sectors. However, their widespread adoption is hindered by inherently weak interfacial bonding, primarily due to the poor surface wettability and unfavorable surface characteristics of natural bamboo. This study presents an innovative fungal-enzymatic pretreatment strategy by inducing the white-rot fungus *Trametes versicolor* (*T. versicolor*) to secrete highly active laccase, which achieves partial depolymerization and modification of lignin, thereby regulating and controlling bamboo surface chemistry and morphology to strengthen interfacial adhesion. This aqueous-based process facilitated the targeted removal of 6.88% lignin and 9.43% hemicellulose, promoted a more crystalline cellulose framework, enhanced the surface wettability, and created a porous microstructure favorable for resin infiltration. Moreover, this lignin depolymerization generated more active functional groups, which contributed to the formation of stronger interfacial bonding in subsequent bamboo composites. As a result, the interfacial bonding of composites was substantially improved, translating into remarkable mechanical enhancements: tensile strength surged by approximately 38.38% and adhesive bond strength increased by about 20.66%. This work demonstrates an aqueous-based biological pretreatment strategy that operates without harsh chemicals, offering a potentially more sustainable route for the development of high-performance bamboo biocomposites for structural applications.

## Graphical abstract

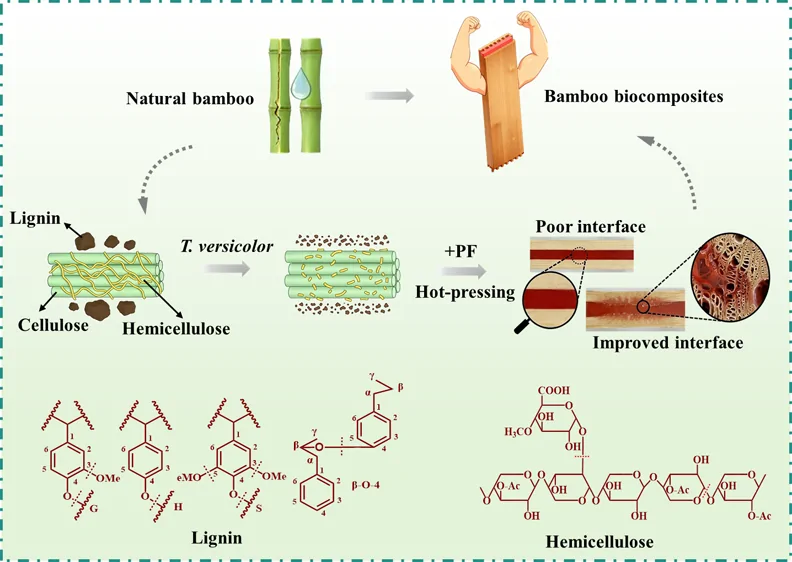

## Introduction

The escalating environmental crises and the urgent need for sustainable development have intensified the pursuit of biobased alternatives to conventional petroleum-based materials.^1^ In this context, biomass composite materials have garnered significant interest across multiple industries owing to their carbon sequestration capacity,^2^ renewability, abundance, and favorable mechanical properties.^3^ Among various biomass resources, bamboo stands out as a particularly promising candidate for next-generation biocomposites owing to its rapid growth rate, remarkable strength-to-weight ratio, and inherent sustainability.^4–6^ However, the widespread adoption of bamboo-based composites is significantly hindered by the inherently poor interfacial compatibility between hydrophilic bamboo fibers and commonly used hydrophobic polymer matrices, which often lead to inefficient stress transfer and compromised mechanical performance.^7,8^

To enhance interfacial adhesion, a range of pretreatment methods have been explored. Conventional physical and chemical approaches, while effective to some extent, are often criticized for their intensive energy consumption, generation of chemical waste, and potential degradation of the natural fiber structure, thereby compromising their eco-friendly profile and economic viability.^9–13^ In contrast, biological modification using fungi and enzymes has emerged as a promising, mild, and energy-efficient alternative for the surface treatment of lignocellulosic biomass.^14^

The interfacial performance of bamboo biocomposites is critically influenced by their chemical composition. Lignin, despite contributing to structural rigidity, forms a hydrophobic and recalcitrant barrier that impedes polymer infiltration and weakens interfacial bonding.^15^ Previous studies have indicated that the selective modification or partial removal of hemicellulose and lignin via pretreatment can significantly improve interfacial adhesion and mechanical strength.^16,17^ Fungal and enzymatic pretreatments offer a targeted strategy by leveraging specific enzymes, such as laccase and peroxidases, to modify lignin macromolecules selectively. These enzymes facilitate the cleavage of β-*O*-4 linkages and oxidation of phenolic subunits, leading to lignin depolymerization while largely preserving cellulose integrity.^18^ This process not only increases surface porosity to promote mechanical interlocking with the resin but also enhances chemical bonding by exposing more reactive sites. Specifically, enzymatic modification triggers the cleavage of methoxyl groups and β-*O*-4 ether bonds within the lignin framework, generating active phenolic and aliphatic hydroxyl groups. These liberated active sites undergo polycondensation reactions with phenolic resins to form stable methylene bridges, thereby substantially enhancing interfacial strength.^19^ Notably, white-rot fungi like *Trametes versicolor* (*T. versicolor*) possess a complete enzymatic arsenal for selective lignin degradation, making them ideal agents for this purpose. Our previous work confirmed the superior selectivity of *T. versicolor* for depolymerizing lignin in Dendrocalamus sinicus (*D. sinicus*).^45^

Despite its potential, the widespread application of fungal pretreatment is challenged by the need for precise control over biological processes. The complex interactions between fungi and enzymes and lignocellulose necessitate a careful selection of fungal species and a deeper understanding of the degradation mechanisms to achieve the desired selective modification without excessive degradation. Critically, the effectiveness of fungal pretreatment heavily relies on the efficient production of lignin-degrading enzymes, particularly laccase, which plays a pivotal role in modifying the hydrophobicity of the lignin barrier. However, natural fungal strains often exhibit low and unstable laccase yields under standard cultivation conditions, which significantly limits their practical application. Therefore, developing strategies to induce and enhance laccase production efficiently is essential to realizing the full potential of fungal pretreatment for high-performance bamboo biocomposites.

This study establishes a mild, ambient-condition, and potentially sustainable strategy for bamboo biomodification, based on a synergistic fungal-enzymatic pretreatment with *T. versicolor* that enhances laccase activity through the use of Tween 80 and optimized culture conditions. The pretreatment is designed to selectively modify lignin and partially remove hemicellulose, thereby increasing the reactivity of modified lignin and enhancing the resin permeability and interfacial bonding of pretreated bamboo to improve the mechanical properties of the composites. This work elucidates the underlying biological macromolecule–enzyme interactions and structural variations in bamboo, establishing a direct link between these micro/macromolecular changes and the improved performance.This biological approach presents a viable route under mild aqueous conditions for the fabrication of high-performance lignocellulosic biocomposites.

## Experiment Section

### Materials

The raw bamboo utilized in this study, *D. sinicus* (*D. sinicus*), was harvested from Lincang, Yunnan province of China, and was cut into bamboo strips with dimensions of 70 mm (longitudinal) × 10 mm (radial) × 1 mm (tangential). The white-rot fungus *T. versicolor* employed in the research was supplied from the China Forestry Microbial Species Preservation and Management Center. The chemical reagents included Tween-80 (CAS: 9005–65–6, 98% purity), KH_2_PO_4_ (CAS: 7778–77–0, 99% purity), MgSO_4_·7H_2_O (CAS: 10034–99–8, 99% purity), ammonium tartrate (CAS: 3164–29–2, 98% purity), carboxy–methylcellulose (CAS: 9004–32–4, 98% purity), Avicel (CAS: 9004–34–6, 94% purity), xylan (CAS: 9014–63–5, 90% purity), starch (CAS: 9005–25–8, 98% purity), pectinase (CAS: 9032–75–1, 98% purity), MnSO_4_ (CAS: 10034–96–5, ≥99% purity), veratryl alcohol (CAS: 93–03–8, 98% purity), ABTS (CAS: 30931–67–0, 98% purity), glyoxal (CAS: 107–22–2, 40% purity), and urea (CAS: 57–13–6, 99% purity). All these reagents were purchased from Sinopharm Chemical Reagent Co., Ltd. and were used without further purification.

### Fungal Pretreatment

First, 25 bamboo strips and 10 g of bamboo powder were placed into a 500 mL Erlenmeyer flask, followed by the addition of 3 g of dipotassium hydrogen phosphate (K_2_HPO_4_), 1.5 g of magnesium sulfate heptahydrate (MgSO_4_·7H_2_O), 0.84 g of ammonium tartrate, and 70 mL of trace element solution, ensuring thorough mixing and dissolution. After the sterilization at 121 °C for 1 h, the white-rot fungus was aseptically transferred into the flask and incubated at 28 °C with agitation at 180 rpm for cultivation periods of 10, 20, and 30 days, respectively. On the fourth day of cultivation, Tween-80 was supplemented into the culture as an inducer to enhance the secretory enzyme activity.

### Preparation of the Bamboo Biocomposites

Both control and fungal-pretreated bamboo samples were rinsed three times with deionized water and dried. A phenolic resin (PF resin) (solid content: 52%, viscosity: 475 mPa·s) was applied at a spread rate of 160 g/m^2^. Three bamboo strips were then assembled into a parallel-laminated structure and mechanically pressed under conditions of 150 °C and 5 MPa for 10 min to produce high-performance bamboo-based biocomposites.

### Enzymatic Activity Assay

In this study, the activities of oxidative and hydrolytic enzymes were systematically evaluated using spectrophotometric methods. During the pretreatment process, the culture supernatants were collected every 5 days for enzymatic activity assays. Laccase activity was assayed in sodium acetate-acetic acid buffer containing the crude enzyme extract, using ABTS (2,2′-azino-bis(3-ethylbenzothiazoline-6-sulfonic acid)) as the substrate, by monitoring the absorbance at 420 nm. Manganese peroxidase (MnP) and lignin peroxidase (LiP) activities were determined using MnSO_4_ and veratryl alcohol as substrates in the presence of H_2_O_2_, monitored by the absorbance changes at 240 and 310 nm, respectively.^20^ Hydrolase activities, including endoglucanase, exoglucanase, xylanase, amylase, and pectinase, were quantified using the 3,5-dinitrosalicylic acid (DNS) method with specific substrates (carboxymethyl cellulose, microcrystalline cellulose, xylan, starch, and pectin), through monitoring the absorbance at 540 nm.^21^ All assays were conducted in triplicate.

### Material Properties and Characterization

The effect of fungal treatment on the physical properties of bamboo was investigated by measuring mass loss rate and density. Samples pretreated for 10, 20, and 30 days were retrieved, respectively, rinsed with water, and oven-dried to constant weight at 103 ± 3 °C. Mass loss at each interval was calculated based on the dry weights of both control and fungal-pretreated samples. According to the Chinese National Standard GB/T 17657–2022, air-dried density was determined using defect-free specimens with dimensions of 70 mm (longitudinal section) × 10 mm (transverse section) × 1 mm (radial section).

To assess the surface wettability of both control and fungal pretreated samples, water contact angle measurements were conducted to characterize the interfacial properties. The surface of control and biomodified samples was polished to a smooth surface, and changes in surface wettability were analyzed using a contact angle meter (JC 2000 D3 R) with six repetitions for each group.

The crystalline phase was carried out by X-ray diffraction (XRD, Rigaku Miniflex 600) with a scanning range from 5° to 40° at a scanning rate of 8°/min and a step size of 0.05°. The crystallinity index (CrI) was calculated as follows

1 $$CrI=\frac{I_{002}-I_{am}}{I_{002}}\times 100\%$$

CrI represents the relative crystallinity, *I*_002_ is the maximum intensity of the diffraction angle of the (002) lattice, representing the diffraction intensity of the crystal region, and *I*_am_ represents the scattering intensity of the amorphous background.

Fourier transform infrared spectroscopy (FTIR, Nicolet 6700, Thermo Fisher, USA) was utilized to analyze the functional groups of the samples by the KBr compression method. The mass ratio of KBr to sample was 100:1. Dried bamboo powder, sieved through a 60-mesh screen, was subjected to triplicate measurements, with spectral data acquisition over the 500–4000 cm^–1^ wavenumber range.

The major chemical components of bamboo were determined according to the National Renewable Energy Laboratory (NREL) method. Samples were initially hydrolyzed by 72% cold sulfuric acid (25 °C, 2 h), followed by dilution to 3% sulfuric acid concentration and autoclaved (121 °C, 1 h) to complete hydrolysis. The residue after filtration was regarded as Klaxon lignin. The acid-soluble lignin in the hydrolysate was then quantified by ultraviolet spectrophotometry.

Preparation of cellulose hydrolase lignin (DEL): bamboo powder was hydrolyzed by cellulase enzyme (50 °C, 150 rpm, 48 h), followed by repeated washing with hot water to remove hydrolyzed carbohydrate residues, followed by centrifuging, and freeze-drying, repeated twice to obtain DEL samples. The biotransformation of lignin structure in fungal-pretreated bamboo was investigated using the two-dimensional heteronuclear single quantum coherence (2D-HSQC) NMR technique. During the experiment, lignin samples were dissolved in deuterated dimethyl sulfoxide (DMSO-*d*_6_) to form a gel-like test system for analysis. The 2D NMR spectra were acquired using an AVANCE NEO 500 MHz spectrometer at 25 °C.

### Microstructural Morphology Observation

Scanning electron microscopy (SEM, ZEISS Sigma 300, Germany) and an optical microscope (OLYMPUS IX71, Japan) were used to observe changes in the micromorphological structures of bamboo and the interfacial structure of bamboo biocomposites.

### Mechanical Performance Evaluation

The mechanical properties of natural bamboo and fungal-pretreated bamboo were tested by a universal testing machine (DR-502 A, Dongri, China). For each test group, eight replicate specimens were prepared and tested at a speed of 5 mm/min. Tensile properties of bamboo sheets and bamboo biocomposites were measured in accordance with Chinese National Standard GB/T 7124–2008. The specimens were prepared with a length of 70 mm, a width of 10 mm, and a thickness of *t* mm (along the radial direction) and featured a dumbbell-shaped gauge section with a narrowed width of 1 mm. Additionally, dry bonding strength was characterized, defined as the interfacial adhesion performance between the adhesive and the bamboo, with a standardized bond area of 8.75 mm × 10 mm. The specimen specifications are illustrated in Figure 1. The details of the preparation of the bamboo biocomposites are shown in Figure 2.

**Figure 1:**
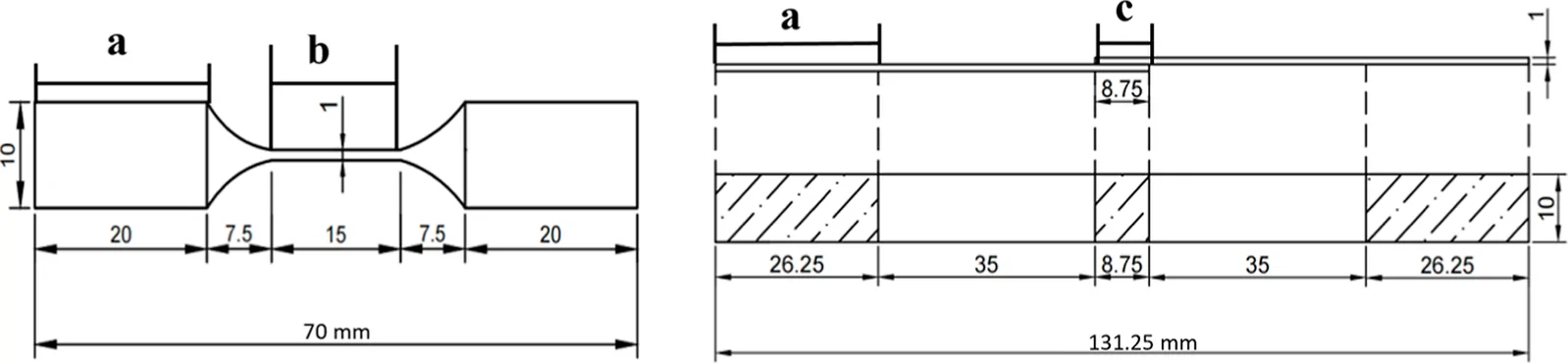
Specimen specification for tensile strength (left) and bonding strength (right) tests. Note: a is the clamping position for the tensile and bonding test fixture; b is the pulling position for the tensile test, and c is the bonding area section. All numerical values in the figure are given in mm.

**Figure 2:**
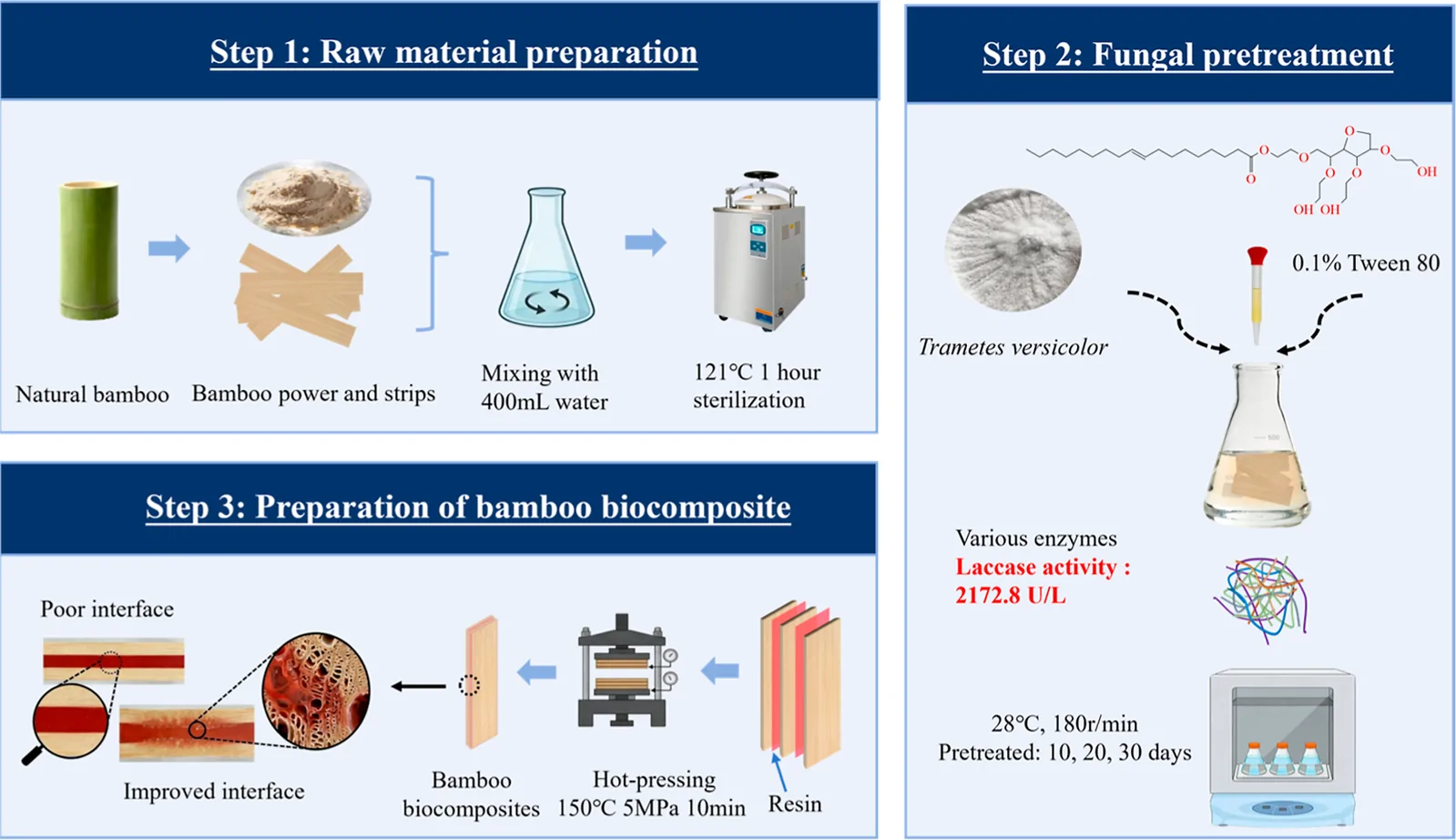
Fabrication process of biocomposites based on fungal-enzymatic bamboo modification.

## Results and Discussion

### Programmed Enzyme Secretion and Controlled Biomodification

The efficient lignin degradation capability of white-rot fungi primarily relies on their extracellular enzyme system, in which laccase, manganese peroxidase, and lignin peroxidase serve as the key enzymes responsible for lignin breakdown. To quantitatively assess the intensity of the white-rot fungal pretreatment, both the enzymatic activities secreted by *T. versicolor* alongside the mass loss rate of bamboo strips throughout the pretreatment process were measured and analyzed.

### Selective Activation of Lignin-Oxidizing Enzymes

To achieve efficient and targeted depolymerization and modification of lignin by white-rot fungus, this study developed a high-efficiency induction system to promote the secretion of highly active lignin-oxidizing enzymes. As depicted in Figure 3a, laccase activity increased nearly 3-fold from 5 to 30 days, reaching a peak of 2175.83 U/L on day 20, although a slight decline was observed by day 30, and laccase activity remained high at 2026.89 U/L. Table 1 compares the laccase activity induced in this study with previously reported values under different culture conditions. When white-rot fungi were cultivated in different media (PDB, corn silage, and multifactorial induction), the laccase activities reached 1291.7 U/L, 1539.4 U/L, and 1113.8 U/L, respectively. In comparison, the laccase activity induced in our study (2175.83 U/L) demonstrates a substantial improvement over those of the previously reported values. The significant enhancement in laccase activity can be attributed to the synergistic effects of trace elements such as Cu^2+^, which facilitate the construction of its active center and directly participate in the catalytic reaction to improve its efficiency,^25^ ample carbon sources that supply essential energy, and Tween 80 that markedly improves cellular membrane permeability. This coordinated promotion significantly increased the efficiency of the lignin modification. Laccase oxidizes phenolic hydroxyl groups in lignin, generating phenoxy radicals that initiate the depolymerization of phenolic lignin subunits.^26^ Concurrently, more functional groups such as hydroxyl and carboxyl are exposed in the depolymerized products, which could form hydrogen or covalent bonds with adhesives more easily, thereby enhancing the interfacial adhesion with the polymer matrix. In contrast, the activities of manganese peroxidase and lignin peroxidase remained relatively low, averaging only 46.22 U/L and 25.45 U/L, respectively, both lower than those in the control groups. These findings suggest that the modification of lignin during subsequent fungal pretreatment stages is primarily mediated by laccases.

**Figure 3:**
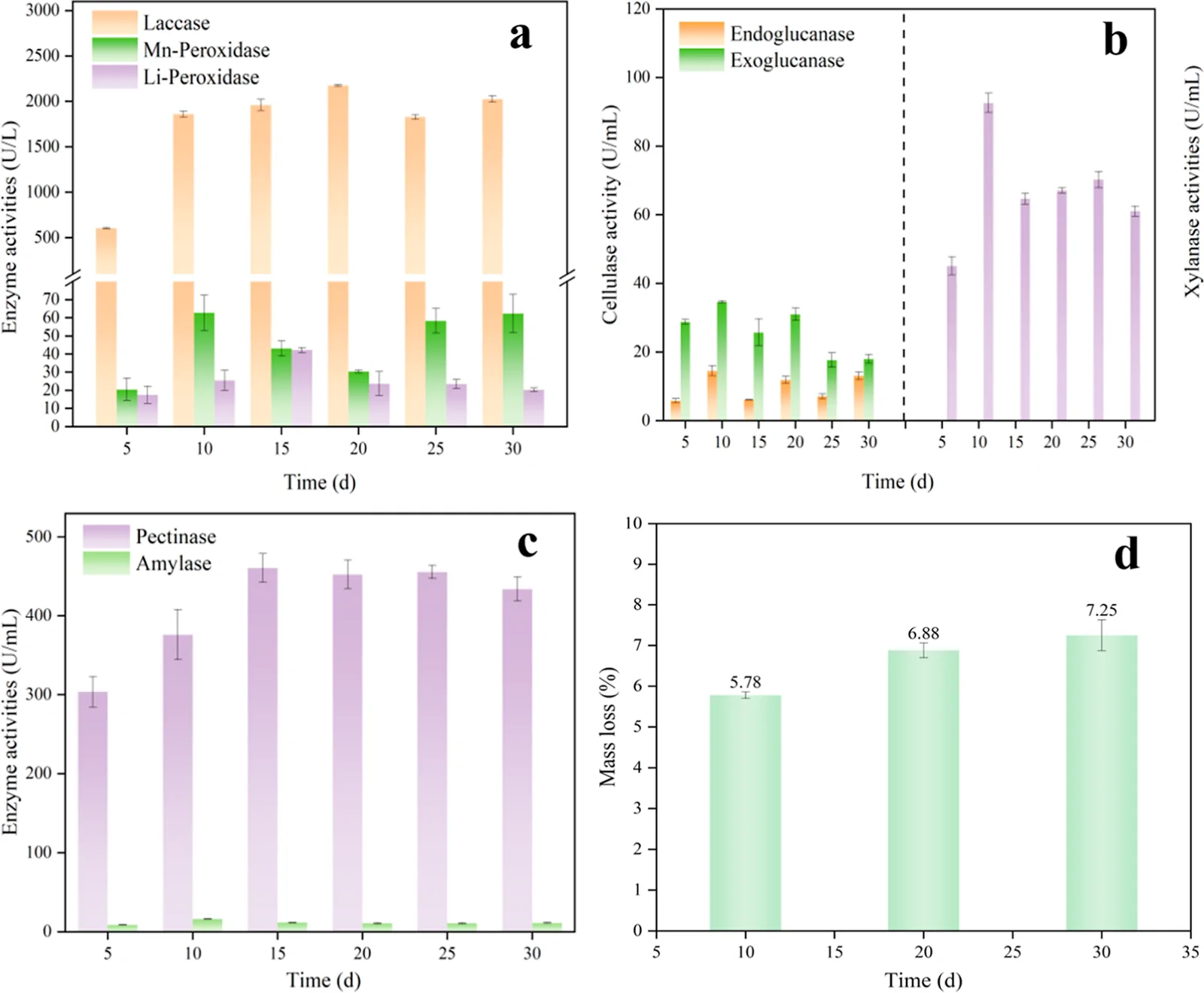
Lignocellulolytic enzyme activities and mass loss at different pretreatment stages: (a) lignin oxidases, (b) cellulase and xylanase, (c) pectinase and hydrolases, (d) mass loss rate.

**Table 1:** Comparison of the Laccase Activity of the Bamboo Pretreatment Process of this Work with Previous Research

strains	culture medium/substrate	lac (U/L) * ABTS	Ref
T. versicolor	potato dextrose broth (PDB)	1291.7	^22^
	corn silage	1539.4	^23^
	glucose 2 g, KH_2_PO_4_ 0.2 g, FeSO_4_ ·7H_2_O 0.035 g, MgSO_4_·7H_2_O 0.05 g, NH_4_Cl 0.5 g, CaCl_2_ 0.0755 g	1113.8	^24^
	tween-80 0.1%, KH_2_PO_4_ 3g, MgSO_4_·7H_2_O 1.5 g, ammonium tartrate 0.84g, trace elements 70 mL, bamboo powder 10g	2172.8	this work

### Synergistic Enzyme Activities for Targeted Modification

The hydrolytic enzyme activities are presented in Figure 3b,c. Pectinase activity remained consistently high throughout the entire pretreatment process, indicating that pectin served as one of the nutrient sources during the initial colonization by white-rot fungi. Following the preliminary degradation and removal of pectin, hemicellulose was progressively exposed. Xylanase activity peaked at 92.62 U/mL on day 10 of pretreatment and subsequently declined and stabilized. This trend can be ascribed to the inherently loose and readily degradable structure of hemicellulose, which is quickly exposed and decomposed during the initial phase. This phenomenon indicates that white-rot fungi secrete enzymes to degrade a minor fraction of polysaccharides, thereby obtaining essential nutrients in the early stages of pretreatment. In contrast, cellulase activity remained low during the entire process, never exceeding 40 U/mL, an effect attributed to the encapsulation of cellulose by lignin and hemicellulose, the gradual depolymerization of these components only slowly exposing the cellulose to enzymatic action. Consequently, exocellulases showed limited accessibility to cellulose chain ends. The consistently higher activity of exocellulases compared with endocellulases throughout the process indicates a fungal preference for attacking terminal glucose units, facilitating their depolymerization into monosaccharides.^27^ These observations collectively demonstrate the characteristic selective delignification mode of white-rot fungi, where metabolic pathways are directionally induced to preferentially decompose lignin rather than nonspecifically degrading all cell wall components.

The selective removal of amorphous components (e.g., pectin, starch, and hemicellulose) increases substrate crystallinity by enhancing the relative proportion and surface accessibility of the crystalline regions. This structural evolution, coupled with the consistently low cellulase activity, which facilitates efficient lignin modification by white-rot fungi while minimizing cellulose degradation,^28^ indicates that the fungal modification process was predominantly driven by lignin-oxidizing enzymes, particularly laccase.

### Controlled Mass Loss and Quantitative Evaluation

During the bamboo pretreatment process, the mass loss was monitored at various stages to quantify the extent of component degradation and avoid excessive decomposition that could compromise the properties of the resulting biocomposites. As illustrated in Figure 3d, the mass loss of bamboo samples exhibited an increasing trend with prolonged exposure to white-rot fungus (10 days: 5.78%, 20 days: 6.88%, and 30 days: 7.25%), with a decelerated rate in the mid-to-late stages. Notably, the majority of the mass loss occurred during the initial phase. This is attributed to the fungal priority for readily metabolizable carbohydrates on bamboo surfaces. Unlike macromolecular cellulose, which resists immediate degradation, pectin has a lower-molecular weight and was preferentially targeted by consistently high pectinase activity (approximately 400 U/mL), which significantly exceeded the cellulase activity. This led to the rapid disintegration of the middle lamella and consequent mass loss. As fungal colonization progressed into the bamboo interior, the synergistic action of high laccase and hemicellulase activities drove the rapid degradation of lignin and hemicellulose, resulting in a significant increase in the mass loss rate. However, the increase in mass loss decelerated due to declining enzymatic activities and limited carbon accessibility imposed by the recalcitrant lignin-carbohydrate complexes (LCCs) in the secondary cell wall in the later stage.^29^ Throughout the entire pretreatment process, the mass loss rate remained below 10%, indicating that no excessive degradation occurred. This observation aligns with the enzymatic activity profiles, which confirmed the partial depolymerization and removal of lignin and hemicellulose mediated by the highly active laccase and xylanase during pretreatment.

### Molecular Reconfiguration of the Bamboo Cell Wall

#### Evolution of Chemical Constituents and Crystalline Architecture

##### FTIR Evidence of Lignin Modification

To evaluate the alterations in the chemical structure of pretreated bamboo lignocellulose, the FTIR spectra for both natural and fungal-pretreated bamboo are illustrated in Figure 4a. The absorption peak at 1735 cm^–1^ corresponds to the C=O stretching and C–O vibrations of acetyl groups in hemicellulose, while the bands at 1600 cm^–1^, 1245 cm^–1^, and 1430 cm^–1^ are attributed to the C=C stretching vibration of the aromatic skeleton, the asymmetric bending vibration of CH_3_ groups, and the combined CH_2_ aromatic skeletal vibrations (lignin) and C–H in-plane deformation (cellulose), respectively.^30^ Compared with natural bamboo, the peak intensities at 1735 cm^–1^, 1600 cm^–1^, and 1245 cm^–1^ in pretreated bamboo decreased to varying degrees with extended treatment time, indicating partial depolymerization of lignin and hemicellulose mediated by lignin-oxidizing enzymes and xylanase, which is consistent with the high laccase activity detected in the enzymatic assays during the pretreatment. Meanwhile, the stable band at 1430 cm^–1^ suggested the integrity of the crystalline cellulose framework was well preserved, which was attributed to the low cellulase activity secreted by the white-rot fungus. This specificity, resulting from the low cellulase activity, preserved the mechanical framework of bamboo and created a more reactive surface for superior adhesive bonding.

**Figure 4:**
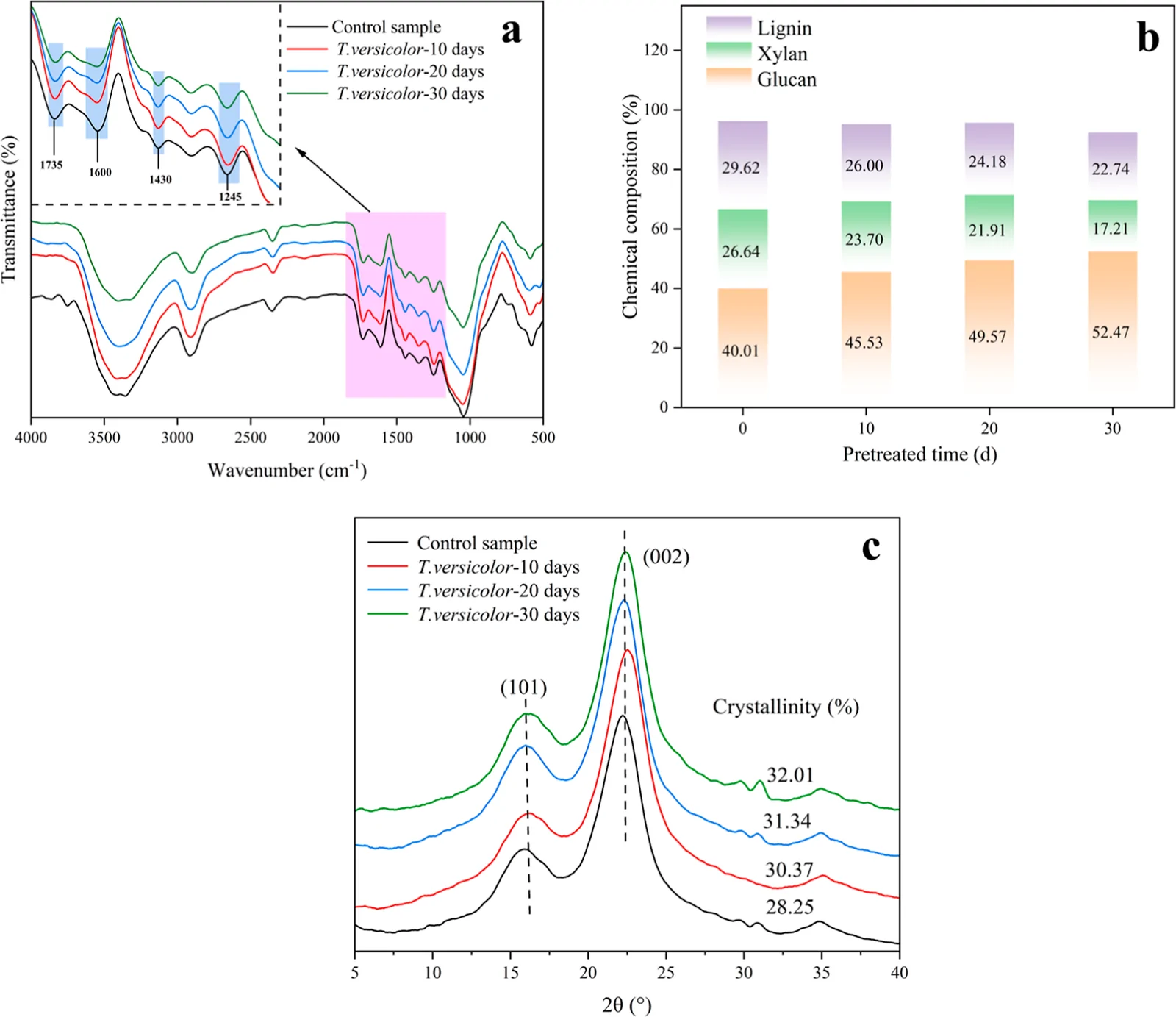
Chemical and compositional structural analysis of bamboo samples: (a) FTIR spectra, (b) chemical compositions, (c) XRD patterns.

##### Selective Delignification and Building a Cellulose-Rich Scaffold

The chemical composition of both control and modified bamboo samples at different stages was measured by acid hydrolysis, as summarized in Figure 4b. The results indicated that the contents of hemicellulose and lignin were significantly reduced after treatment (by 9.43% and 6.88%, respectively), which is consistent with the observed mass loss and changes in FT-IR spectra. These reductions further demonstrate the partial removal of lignin and hemicellulose facilitated by the action of highly active lignocellulolytic enzymes, particularly laccase.

In general, the stable structure of lignin necessitates the secretion of highly active laccase by fungi to enable its targeted depolymerization or delignification. This result also confirms the successful establishment of an efficient enzyme secretion system for pretreatment. Hemicellulose, characterized by its inherently low polymerization and loose structure in its native state, is more susceptible to degradation.^31^ Moreover, lignin and hemicellulose are interconnected via ester and ether linkages, thus, the depolymerization of lignin consequently leads to partial breakdown of hemicellulose.^32^ Notably, the relative content of cellulose increased, which is pivotal for the subsequent fabrication of bamboo biocomposites as highly crystalline cellulose imparts excellent mechanical properties to the resulting materials.

##### XRD Insights into Crystalline Cellulose Development

FTIR and compositional analyses, which indicated a decrease in lignin and hemicellulose along with a relative increase in cellulose, necessarily led to the rise in crystallinity of *D. sinicus* as pretreatment time extended. The supramolecular structure of biomass materials can be analyzed by using XRD techniques. As presented in Figure 4c, compared with the control sample, the (101) and (002) diffraction peaks of bamboo treated with white-rot fungus for different durations exhibited differential increases in intensity, while their peak positions remained largely unchanged. This observation suggests that the cellulose crystalline structure remained unaltered after pretreatment, maintaining the native cellulose I allomorph. Such findings demonstrate that the fungal and enzymatic treatments preserve the crystalline integrity of cellulose, thereby ensuring it continues providing structural support to the cell wall.^33^

The calculated results indicated that the CrI of the fungal-pretreated sample was higher than that of the control (increasing from 28.25% to 32.01%). This enhancement in crystallinity can be attributed to the selective removal of amorphous hemicellulose and lignin facilitated by fungal-enzymatic activity during pretreatment.^34,35^ Concurrently, the exposed hydroxyl groups on amorphous microfibrils formed hydrogen bonds with those on the surfaces of crystalline regions,^36^ thereby contributing to the overall crystallinity improvement, which is beneficial for enhancing the mechanical properties of the resulting biocomposites.

### 2D-HSQC Mapping of Lignin Structural Changes

To gain further insight into the transformation of lignin’s molecular structure during fungal pretreatment, we recorded the two-dimensional (2D) NMR spectra of both natural bamboo and modified bamboo. Lignin is composed of phenylpropane monomeric units primarily connected by ether bonds(e.g., β-*O*-4) and carbon–carbon bonds (e.g., β–β or β-5).^37,38^ As illustrated in panels a and b of Figure 5, various substructures were identified in the side-chain region, including methoxyl (OMe), aryl ether (β-*O*-4, A), resinol (B, β–β), and phenylcoumaran (β-5, C), in the aromatic region, the well-resolved signals originated from the aromatic ring protons and allowed for the identification of guaiacyl (G), syringyl (S), and *p*-hydroxyphenyl (H) units. Following pretreatment, a general reduction in signal intensities was observed, particularly in the aryl ether (β-*O*-4, A) and guaiacyl (G) regions, along with a noticeable decrease in methoxyl signals. These changes indicate substantial depolymerization of lignin structural units, predominantly within the side-chain regions, induced by the fungal and enzymatic pretreatment.

**Figure 5:**
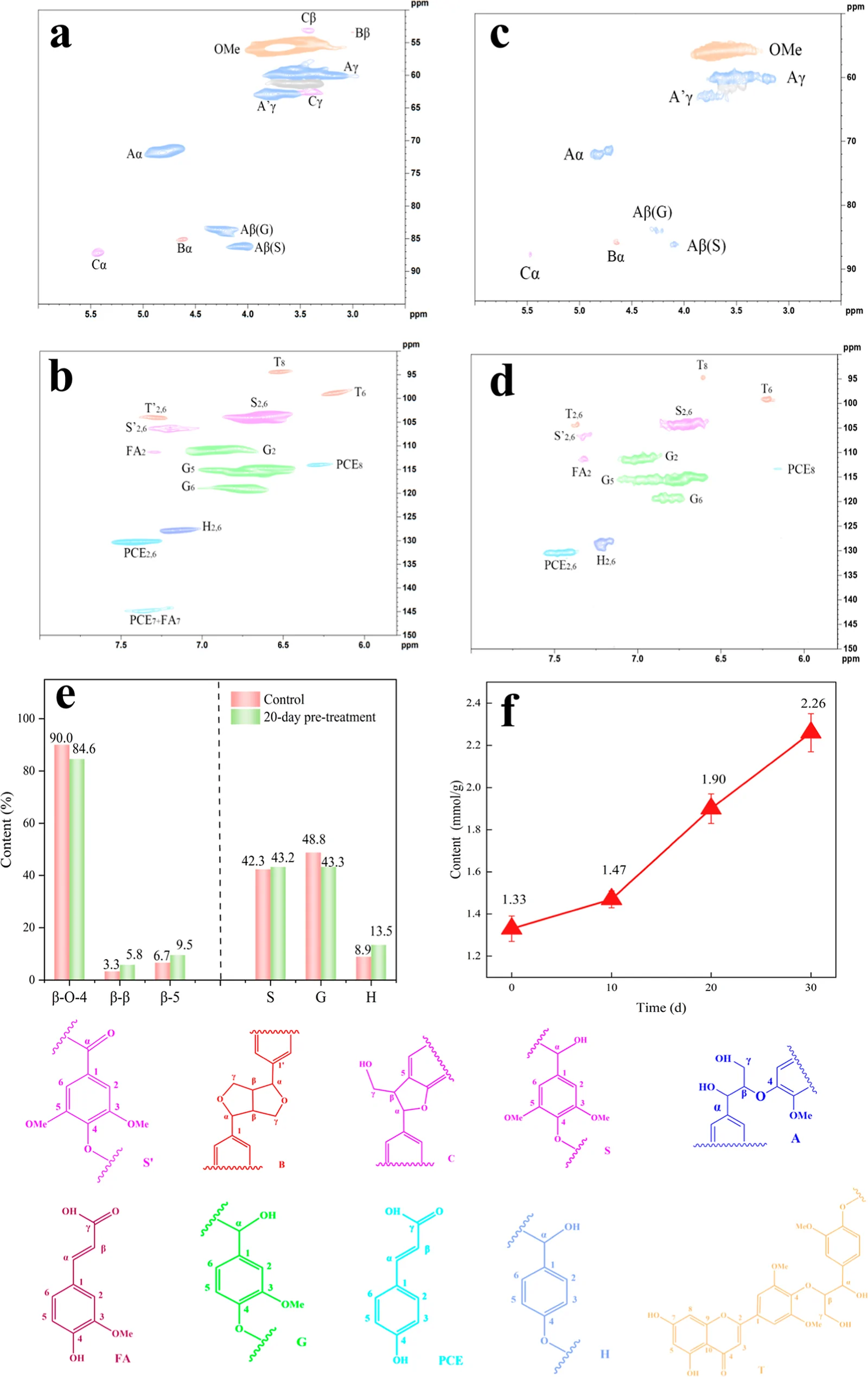
Side-chain and aromatic regions in the 2D-HSQC NMR spectra of the DELs. (a,b) Control sample, (c,d) *T. versicolor*-20 days, (e) NMR quantification of lignin aliphatic structures in natural bamboo and fungal pretreated bamboo, (f) phenolic hydroxyl content of natural and fungal pretreated bamboo.

Quantitative analysis based on spectral integration of major lignin subunits and interunit linkages further corroborated these observations (Figure 4e). Following pretreatment with white-rot fungus, the relative content of S-units and H-units in bamboo lignin increased by 0.9% and 4.6%, respectively, whereas that of G-units decreased by 5.5%. This shift in compositional preference indicated the selective degradation of G-type aromatic units. In the side-chain region, the relative content of β-*O*-4 linkages declined from 90.0% to 84.6%, corresponding to a cleavage extent of 5.4%. Conversely, the relative contents of β–β and β-5 linkages increased. This pattern was primarily attributed to the substantial secretion of laccase during the early stages of fungal treatment. Laccase oxidizes phenolic units, generating phenoxy radicals that destabilize either the C_α_-C_β_ or β-*O*-4 bonds, thereby initiating the cleavage of the β-*O*-4 aryl ether linkages within the lignin backbone. This led to a decrease in their content and a reduction in molecular weight. Although cleavage of carbon–carbon bonds such as β–β and β-5 also occurred, the extensive breakdown of β-*O*-4 ether bonds resulted in a relative increase in their proportion within the residual lignin. Simultaneously, the laccase-catalyzed demethoxylation reaction removes methoxy groups in the form of methanol, resulting in a weakening of the methoxy signal. The lignin depolymerization reaction generates and exposes more active sites on the lignin surface, such as phenolic hydroxyl functional groups (Figure 5f) (control: 1.33 mmol/g; day 10:1.47 mmol/g; day 20:1.90 mmol/g; day 30:2.26 mmol/g). The increase in the number of these active sites could not only enhance the hydrogen bonding between lignin and cellulose but also improve the reactivity of bamboo toward adhesive resins during hot-pressing.^39,40^

These analytical results demonstrate that white-rot fungal pretreatment predominantly targets and cleaves the β-*O*-4 ether bonds, which are the most prevalent linkages within the lignin network. Partial depolymerization occurs in the side-chain structures through laccase-mediated reactions, while the aromatic benzene rings remained largely intact due to their inherent stability. The depolymerization and structural reconfiguration of lignin and hemicellulose underlie the improved porosity and permeability of bamboo, which are essential for subsequent efficient adhesive impregnation and the fabrication of composite materials.

### Structural Changes and Composite Properties

#### Improved Wettability and Pore Structure

The contact angle serves as a key indicator of surface wettability, with smaller angles indicative of greater hydrophilicity. Improving the wettability of lignocellulosic biomass effectively enhances the adhesive strength between the substrate and adhesives.^41,42^ As depicted in Figure 6a, the contact angle gradually decreased with extended treatment time, declining from 104.23° in the control group (day 0) to 77.82° on day 30, directly confirming a significant improvement in wettability. This change can be primarily attributed to the selective degradation and removal of lignin, hemicellulose, and pectin, which not only increased surface roughness and porosity but also significantly enhanced interfacial chemical reactivity by exposing hydroxyl groups of cellulose and reactive sites in modified lignin. These modifications promoted the penetration, mechanical interlocking, and chemical bonding of the adhesive, establishing a critical interfacial foundation for the superior bonding strength and performance of the final composite material.

**Figure 6:**
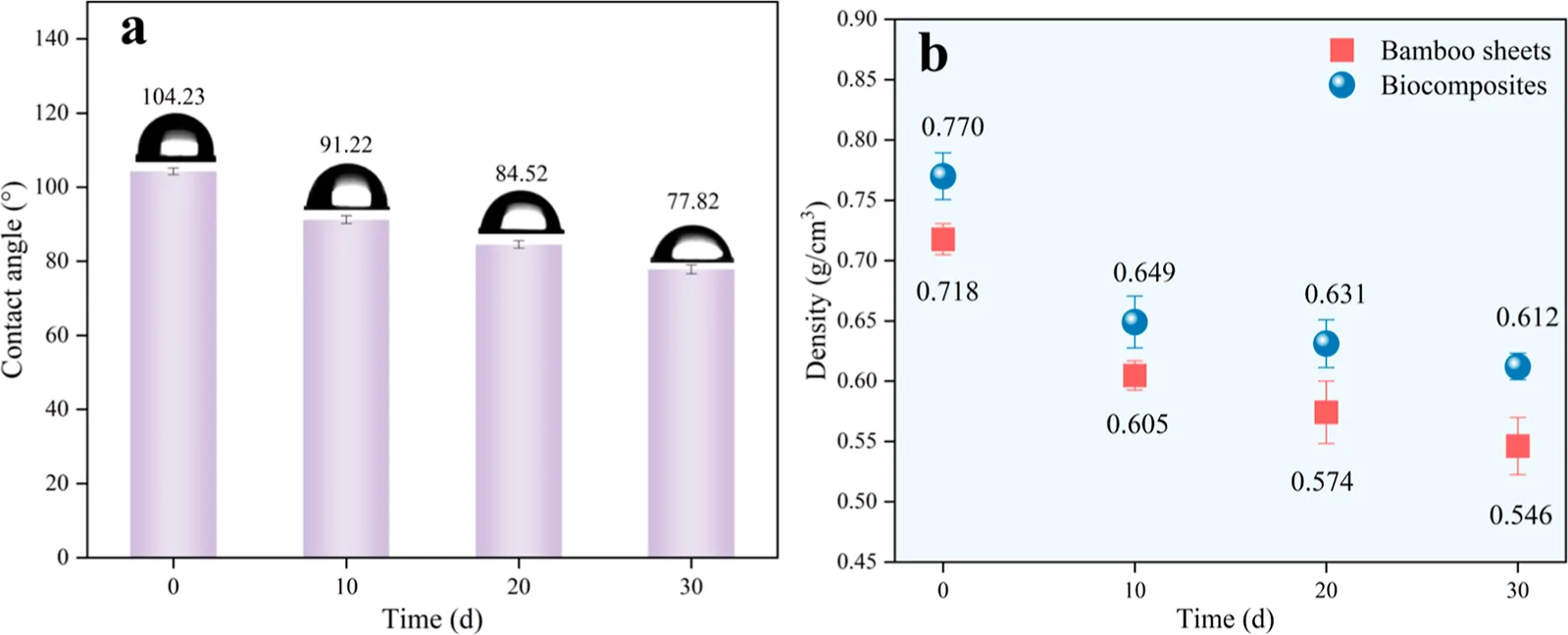
Changes in the physical properties of bamboo and bamboo biocomposites before and after fungal pretreatment: (a) contact angle, (b) density.

White-rot fungal pretreatment significantly modulates the multiscale structure and interfacial properties of bamboo through selective degradation of its components, as intuitively reflected by changes in density. As shown in Figure 6b, the densities of both bamboo strips and bamboo biocomposites exhibited decreasing trends with a prolonged treatment time (bamboo strips decreased from 0.718 g/cm^3^ to 0.546 g/cm^3^, while bamboo biocomposites decreased from 0.770 g/cm^3^ to 0.612 g/cm^3^). This reduction can be primarily attributed to the partial removal of lignin, hemicellulose, and other extractives. The rate of density decrease was higher for bamboo strips than for bamboo biocomposites, indicating that the adhesive contributed to a more-densified structure in the treated bamboo. These density changes intuitively reflect the structural modulation of bamboo induced by white-rot fungal treatment, and the resulting densified microstructure is conducive to the performance enhancements of the bamboo biocomposites.

#### Interfacial Morphology Evolution and Resin Infiltration

Significant alterations in the microscopic morphology of bamboo were observed after white-rot fungal pretreatment, as shown in the micrograph in Figure 7A. The fibers in the control bamboo exhibited a smooth, intact, and densely packed cellular structure. In contrast, following fungal pretreatment, the partial removal of lignin, hemicellulose, and other nutritive components resulted in looser, rougher, and more porous fibers and cell walls within the parenchyma cells, accompanied by noticeable cell shrinkage and collapse. These observations align with previous reports that delignification induces fiber cell separation and displacement, leading to slightly increased porosity and reduced density in treated bamboo,^43^ which is conducive to adhesive penetration and subsequent interfacial bonding with the matrix.

**Figure 7:**
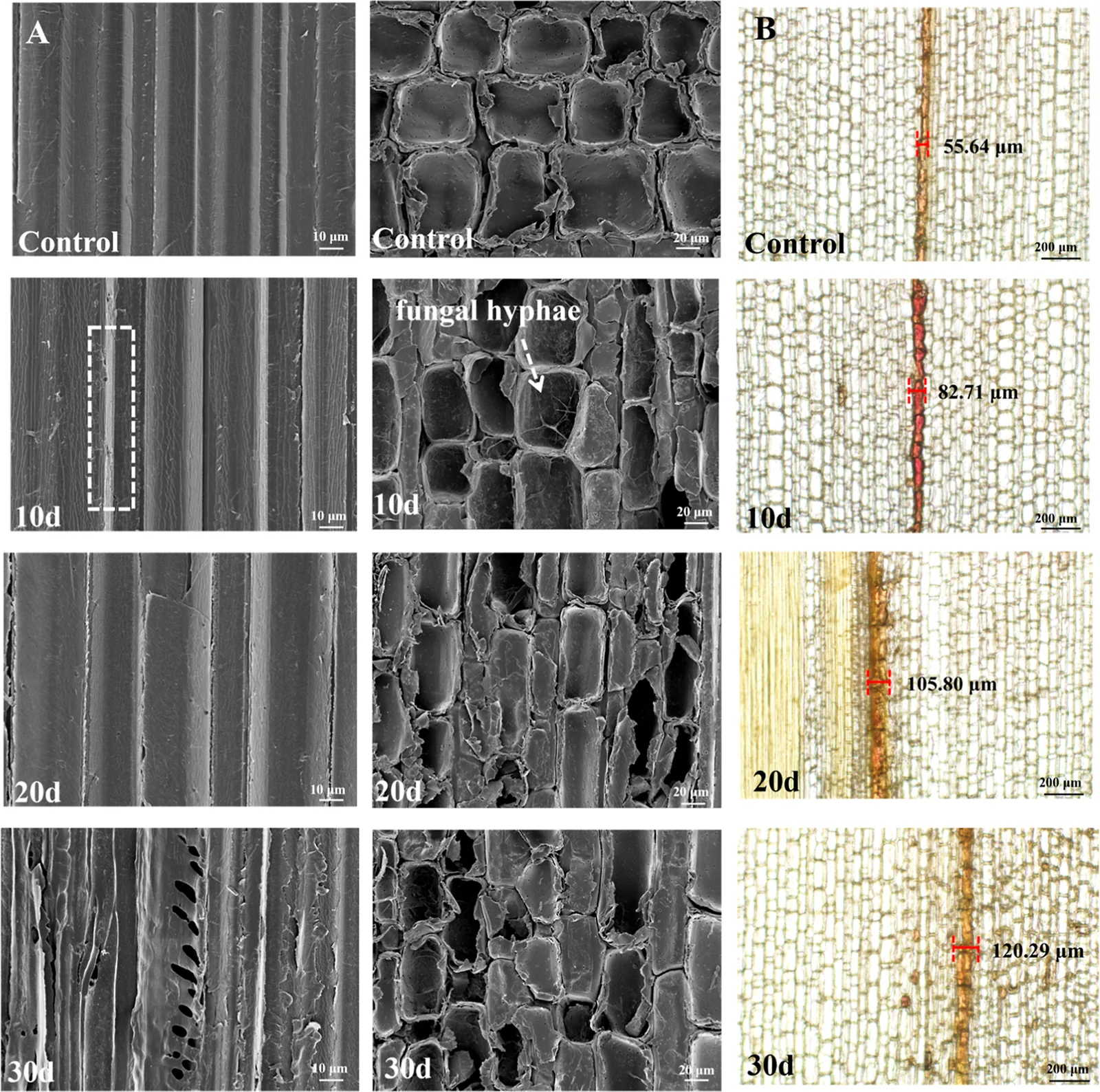
Microstructure of bamboo and bamboo biocomposites. (A) SEM images of bamboo samples before and after pretreatments, (B) optical microscopy images of the bonding interface of bamboo biocomposites. Note: panel (A) shows the microstructure of the bamboo sheets. Panel (B) shows the micromorphology of the bonding interfaces of the bamboo biocomposites. Control refers to the untreated samples, while 10d, 20d, and 30d refer to the fungal-pretreated samples at the respective durations.

The penetration of adhesive within the bamboo biocomposites was visualized using optical microscopy (Figure 7B). Compared with the control group, the adhesive in pretreated samples infiltrated adjacent fibers and cells to varying degrees, with the extent of penetration progressively increasing with longer treatment durations (control: 55.64 μm; 10 days: 82.71 μm; 20 days: 105.80 μm; 30 days: 120.29 μm). The improvement in adhesive distribution and penetration performance can primarily be attributed to the removal of lignin, hemicellulose, and other nutrients, which enhances both the porosity and surface wettability of bamboo. Concurrently, the modification of the fiber surface exposes more small molecular active groups that engage in chemical reactions between the adhesive and the bamboo substrate. This enhanced interfacial reactivity, together with the improved adhesive penetration, facilitates the formation of an interlocked interface. It is worth noting, however, that the relationship between penetration depth and bond performance is not linear, excessive penetration could potentially lead to a starved glue line and weaken the bond. Within the parameters of this study (where bonding strength peaked on day 20 and remained above the control even after a slight decrease by day 30), the achieved degree of penetration remained within the beneficial range, thereby contributing positively to the overall composite performance.

#### Interface-Dominated Mechanical Reinforcement

The mechanical properties of bamboo and its biocomposites are illustrated in Figure 8. Both the bonding strength and tensile strength exhibited an initial increase followed by a decrease with prolonged pretreatment time. The bonding strength increased from 7.89 MPa (0 day) to 9.52 MPa (a 20.66% improvement) before declining to 9.22 MPa (Figure 8a). The tensile strength of bamboo strips increased from 157.13 to 185.92 MPa (an increase of 18.32%), subsequently declining to 177.06 MPa. Meanwhile, the tensile strength of the biocomposites improved from 203.36 to 281.37 MPa (Figure 8b), an enhancement of 38.36%, which is 14.47% higher than that of the control group reported in a previous study,^44^ before eventually falling to 224.60 MPa, a value which, although decreased, remained higher than that of the control. Both the bonding strength and tensile strength reached their maximum values at 20 days of pretreatment, indicating this duration as the optimal window for the modification process. Notably, the bonding strength peaked at 20 days of pretreatment, accompanied by a substantial increase in bamboo failure percentage from 50% at 0 day to 83.33% at 20 days, with the fracture location progressively shifting from the adhesive–bamboo interface toward the bamboo substrate (Figure 8c,d). This observation demonstrates that the interfacial bond strength had exceeded the cohesive strength of the bamboo substrate, meaning that the adhesive layer was no longer the weak point in failure, confirming that the interfacial bonding after pretreatment surpassed the inherent strength of the native bamboo. This further validates the successful enhancement of bamboo bonding interface.

**Figure 8:**
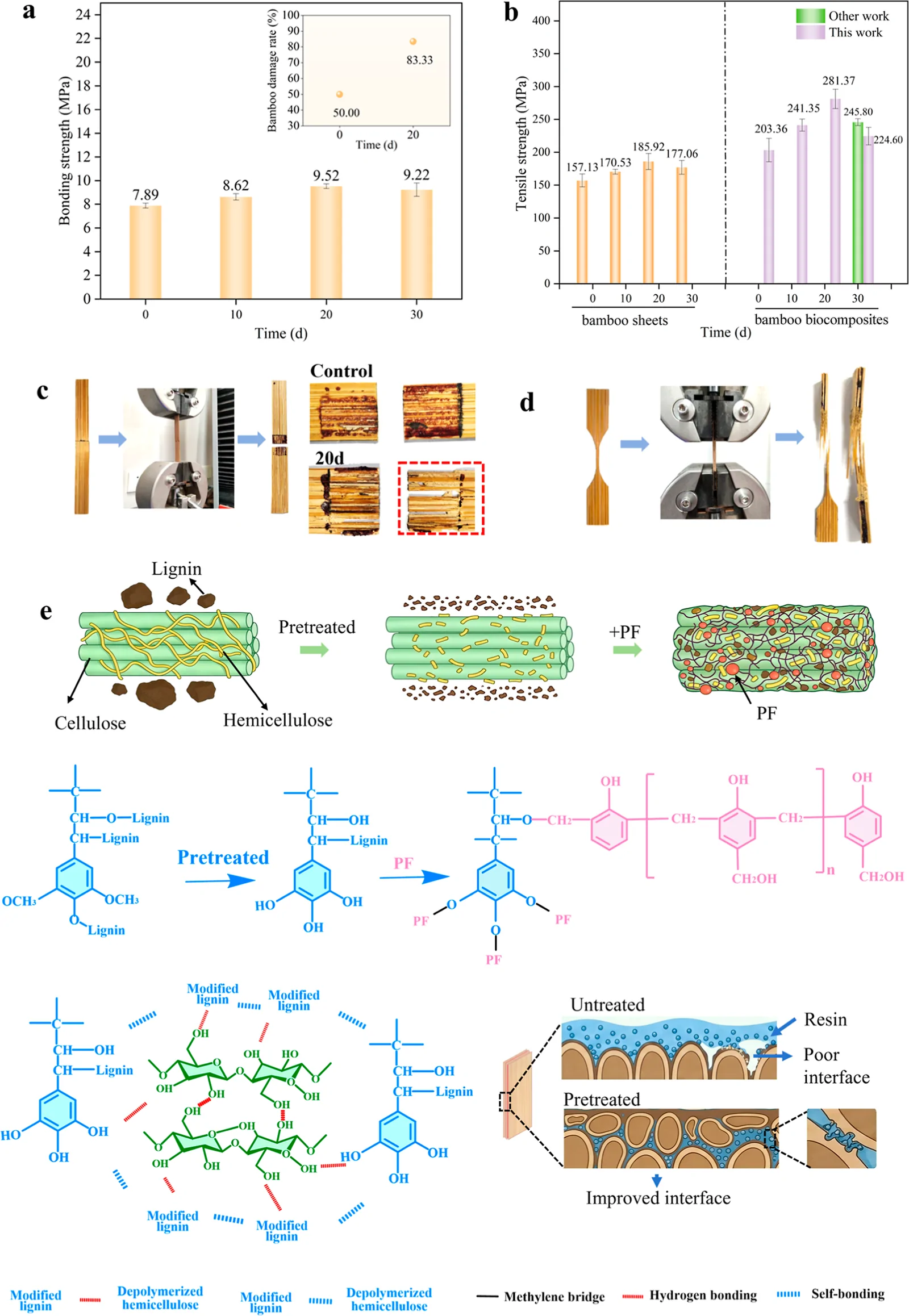
Mechanical properties: (a) bonding strength and damage rate of bamboo, (b) tensile strength (bamboo sheets and biocomposites), (c,d) schematic diagram of bonding strength and tensile strength tests, (e) interfacial reinforcement mechanism of bamboo biocomposites.

The enhancement in mechanical properties can be attributed to several key factors: Compared with natural bamboo, fungal pretreatment induced significant depolymerization and modification of lignin and hemicellulose, increasing the porosity and augmenting the surface wettability of the treated bamboo samples. This enhancement facilitated more effective adhesive distribution and penetration across the bamboo matrix, leading to the formation of denser and more uniform biocomposites. Besides, enzymatic pretreatment further exposed additional reactive groups by modifying lignin, which could strengthen van der Waals forces and hydrogen bonding, a pivotal process during hot pressing.^45,46^ Specifically, NMR spectroscopy confirmed that enzymatic modification triggers the cleavage of methoxyl groups and β-*O*-4 ether bonds within the lignin macromolecular framework. This structural degradation directly leads to an increase in active phenolic hydroxyl group contents. These active sites could participate in the condensation reactions with the PF resin, forming methylene bridges or methylene ether bonds (Figure 8e), thereby chemically linking lignin to the molecules of PF resin.^47^ Meanwhile, the activated lignin exposes additional hydroxyl (−OH) groups, which have the potential to form more stable hydrogen bonds with the surface hydroxyls of cellulose and depolymerized hemicellulose, thereby facilitating the formation of more stable interfacial connections between fibers. Furthermore, within modified lignin molecules, self-condensation reactions may occur, along with co–condensation reactions between modified lignin and depolymerized hemicellulose, leading to the formation of a stable, cross-linked network.

The synergy arising from the mechanical anchors formed by the more effective penetration of the PF resin and the enhanced chemical interactions, including both covalent and hydrogen bonding within the interfacial region, contributes to the improved bonding interface of composites from both physical and chemical standpoints. Consequently, this combination markedly improves interfacial bonding, adhesive strength, and the mechanical performance of bamboo biocomposites.^48,49^ However, when the pretreatment exceeded 20 days, the mechanical properties deteriorated significantly. This decline can be likely attributed to the damage to the bamboo cell wall caused by prolonged biological treatment, which compromises the inherent mechanical integrity of the bamboo.

#### Comparative Sustainability Assessment

To evaluate the proposed fungal-enzymatic pretreatment, we benchmarked it against two widely reported chemical approaches, namely alkali and acid treatment, that are commonly used to modify bamboo for composite fabrication. A comparative assessment of these three methods reveals clear distinctions in their environmental and performance characteristics, as shown in Table 2. Alkali treatment relies on corrosive NaOH, generates alkaline wastewater requiring costly neutralization, and lacks selectivity, damaging the fiber structure. Acid treatment involves chemical input, produces acidic waste, and similarly compromises mechanical properties. Both approaches rely on chemical agents that pose potential environmental concerns under their respective usage conditions. In contrast, the fungal-enzymatic method operates under ambient conditions (28 °C) using biodegradable nutrients without strong acids or alkalis. It exhibits high selectivity, preserving cellulose integrity while preferentially degrading lignin. The water consumption is similar to that of acid treatment and lower than that of the alkali treatment. Wastewater from the fungal process contains enzymes, nutrients and bamboo extractives, partial reuse may be feasible under controlled conditions, but eventual treatment and disposal requirements depend on reuse limits and must be assessed during scale-up. A full life-cycle assessment (LCA) would be required to quantitatively compare the overall environmental footprints of these pretreatment methods. This translates into superior performance: bonding strength reached 9.52 MPa and tensile strength 281.37 MPa, which are 44.9% and 44.2% higher than those from alkali treatment (6.57 and 195.09 MPa), and 111.6% and 47.6% higher than those from acid treatment (4.5 and 190.60 MPa).

**Table 2:** Comparative Sustainability Assessment of Pretreatment Methods for Bamboo Biocomposites

aspect	this study: fungal-enzymatic pretreatment	conventional pretreatment: alkali treatment^50,51^	acid pretreatment^52,53^
processing conditions	temperature: 28 °C (ambient), 10 days, 20 days	1%, 4%, or 7% NaOH solution, 3 h at room temperature	10% citric acid, vacuum/pressure impregnation, cured at 120 °C, 48 h
energy consumption	98 kWh/kg bamboo (10 days); 195 kWh/kg bamboo (20 days) (mainly for incubation)	15 kWh/kg bamboo (drying at 80 °C for 8 h)	142 kWh/kg bamboo (heating for curing at 120 °C)
chemicals used	biodegradable nutrients, no strong acids or alkalis	sodium hydroxide (corrosive)	citric acid (weak organic acid)
water consumption	0.20 m^3^/kg bamboo (medium preparation, 20 days)	0.45 m^3^/kg bamboo (solution preparation + washing for alkali removal)	0.25 m^3^/kg bamboo (Washing after impregnation to remove unreacted chemicals)
wastewater & waste	contains enzymes, nutrients and bamboo extractives; may be reusable for several cycles, but eventually requires treatment	alkaline wastewater requires neutralization	acidic wastewater requires neutralization
cost (bamboo fiber + chemicals)	6 CNY/kg	20 CNY/kg	15 CNY/kg
selectivity	high selectivity (preferentially degrades lignin, preserves cellulose)	low selectivity (nonspecific attack on both lignin and cellulose)	low selectivity (nonspecific attack on both lignin and cellulose)
mechanical properties	bonding strength: 8.62 MPa (10days); 9.52 MPa (20days) tensile strength: 241.35 MPa (10days); 281.37 MPa (20days)	bonding strength: 6.57 MPa tensile strength: 158.20–195.09 MPa; 65.58–140.23 MPa	bonding strength: 4.5 MPa; 0.37 MPa tensile strength: 190.60 MPa

Although the fungal-enzymatic method requires a longer incubation period (20 days, 195 kWh/kg), comparable mechanical properties are achieved after only 10 days (98 kWh/kg), suggesting that energy consumption could be reduced through optimization. The fungal-enzymatic approach avoids high-temperature processing and corrosive reagents, offering a highly selective route to improve bamboo composite performance, and its overall environmental performance should be quantified by LCA in future work.

## Conclusions

A fungus-secreted enzymatic bioconversion for the bamboo interface has been successfully explored, and high-performance bamboo biocomposites have been developed. The principal findings can be summarized as follows.

1) An efficient induction system was established to stimulate the white-rot fungus *T. versicolor* to secrete highly active lignocellulose-degrading enzymes, particularly laccase, enabling selective removal and modification of lignin and hemicellulose. This process not only exposed more reactive sites but also improved the surface wettability and pore structure of bamboo, synergistically enhancing interfacial bonding between the adhesive and bamboo matrix through both improved physical penetration and chemical interactions.
2) Pretreatment for an optimal duration (20 days) significantly improved the mechanical properties of both bamboo strips and the resulting biocomposites. After 20 days of pretreatment, the tensile strength and bonding strength of bamboo strips as well as the tensile strength of bamboo biocomposites reached their maximum values of 185.92 MPa (18.32% increase), 9.52 MPa (20.66% increase), and 281.37 MPa (38.33% increase), respectively, demonstrating the effectiveness of the enzymatic modification process.

In summary, this study demonstrates a fungal-enzymatic pretreatment for fabricating bamboo biocomposites that operates under mild aqueous conditions without high temperatures or corrosive chemicals. This approach enables effective modification of the bamboo surface through targeted depolymerization and modification of lignin, leading to improved interfacial bonding and enhanced mechanical strength. While a full life cycle assessment would be needed to quantitatively establish its overall environmental performance, this biological route offers a highly selective and potentially more sustainable alternative for developing high-performance biobased composites.
